# Applying the Participatory Slow Design Approach to a mHealth Application for Family Caregivers in Pediatric Ear, Nose, and Throat Surgery

**DOI:** 10.3390/healthcare12040442

**Published:** 2024-02-08

**Authors:** Raffaella Dobrina, Anja Starec, Laura Brunelli, Eva Orzan, Chiara De Vita, Livia Bicego, Luca Ronfani, Veronica Castro, Paola Di Rocco, Sara Zanchiello, Margherita Dal Cin, Benedetta Tagliapietra, Michela Cinello, Donatella Fontanot, Tamara Stampalija, Angelo Dante, Cristina Petrucci, Andrea Cassone

**Affiliations:** 1 Healthcare Professions Directorate, Institute for Maternal and Child Health—IRCCS “Burlo Garofolo”, 34137 Trieste, Italy; livia.bicego@burlo.trieste.it (L.B.); andrea.cassone@burlo.trieste.it (A.C.); 2 Area Science Park, 34149 Trieste, Italy; anja.starec@areasciencepark.it (A.S.); chiara.devita@areasciencepark.it (C.D.V.); sara.zanchiello@areasciencepark.it (S.Z.); michela.cinello@areasciencepark.it (M.C.); 3 Department of Medical, Surgical and Health Sciences, University of Trieste, 34127 Trieste, Italy; laura.brunelli@phd.units.it (L.B.); tamara.stampalija@burlo.trieste.it (T.S.); 4 Audiology and Otorhinolaryngology Unit, Institute for Maternal and Child Health—IRCCS “Burlo Garofolo”, 34137 Trieste, Italy; eva.orzan@burlo.trieste.it (E.O.); veronica.castro@burlo.trieste.it (V.C.); 5 Clinical Epidemiology and Public Health Research Unit, Institute for Maternal and Child Health—IRCCS “Burlo Garofolo”, 34137 Trieste, Italy; luca.ronfani@burlo.trieste.it; 6 Surgery Unit, Institute for Maternal and Child Health—IRCCS “Burlo Garofolo”, 34137 Trieste, Italy; paola.dirocco@burlo.trieste.it (P.D.R.); benedetta.tagliapietra@burlo.trieste.it (B.T.); 7 Department of Health Prevention, Azienda Sanitaria Universitaria Giuliano Isontina, 34148 Trieste, Italy; margherita.dalcin@asugi.sanita.fvg.it; 8 Public Relations Office, Institute for Maternal and Child Health—IRCCS “Burlo Garofolo”, 34137 Trieste, Italy; donatella.fontanot@burlo.trieste.it; 9 Unit of Fetal Medicine and Prenatal Diagnosis, Institute for Maternal and Child Health—IRCCS “Burlo Garofolo”, 34137 Trieste, Italy; 10 Department of Health, Life and Environmental Sciences, University of L’Aquila, 67100 L’Aquila, Italy; angelo.dante@univaq.it (A.D.); cristina.petrucci@univaq.it (C.P.)

**Keywords:** mHealth, patient-centered education, participatory design, tonsillectomy, ear, nose, throat surgery, children, perioperative care, family-centered care

## Abstract

Pediatric ear, nose, and throat (ENT) surgery is very common, and its outcomes may improve with family education. In this regard, mobile health (mHealth) applications (apps), which are on the rise due to digital transformation, can be beneficial in healthcare. This study outlines the user-centered design and development of a mHealth app (version 5.15.0) to support family caregivers during the perioperative process of pediatric ENT surgery. Conducted over two years in an Italian maternal and child health hospital (January 2020–May 2022), the study employed a participatory design method based on the Information System Research (ISR) framework and guided by the principles of Slow Medicine. Utilizing the Relevance, Rigor, and Design cycles of the ISR framework, the mHealth app’s content, functionalities, and technical features were defined and developed. A committee of fifteen experts guided the process with input from 25 family caregivers and 24 healthcare providers enrolled in the study. The mHealth app content was structured around five crucial educational moments characterizing the ENT perioperative period, providing evidence-based information on surgical procedures, strategies for preparing children for hospitalization and surgery, pain management, and post-discharge care. The mHealth app featured a function that sends customized notifications to guide caregivers at specific perioperative stages. The development of mHealth apps by implementing a rigorous, participatory, and Slow design process can foster accessible and family-centered information and care in the field of maternal and child health and beyond.

## 1. Introduction

Ear, nose, and throat (ENT) surgery is common in children [1]. However, previous research findings reported that children and their families are unsettled by the experience of the perioperative processes related to this specific type of pediatric surgery. Fear of the unknown can indeed stress the pre-operative period, while pain and other potential complications such as fever, vomiting, limited oral intake, or bleeding may make post-operative management at home challenging [2,3]. Moreover, parental anxiety has been found to worsen children’s perception of pain, perioperative stress, and the recovery process [4,5].

Within this context, it has been shown that preparing children and their families for hospitalization, surgery, and post-operative home management can improve perioperative outcomes [2,6,7]. However, ENT surgeries such as tonsillectomy usually require a short hospital stay, and healthcare providers (HCPs) have little time to provide adequate support and education to families. This places a significant burden on family caregivers, who must take on more responsibility for managing children’s symptoms, such as pain and functional limitations, at home [4].

The use of mobile health (mHealth) applications (apps) in healthcare is increasing and has far-reaching implications for clinical practice, including disease prevention and care as well as health promotion. More specifically, the benefits of mHealth apps have been widely discussed in terms of their potential for implementing health monitoring, supporting patients-HCPs communication, and empowering patients in health decision-making [8]. As a case in point, a recent survey-based pilot study reports that family caregivers of children who underwent tonsillectomy responded positively to postoperative information delivered via smartphone with a mHealth app, preferring this medium to information delivered via paper instructions [9].

Moreover, the COVID-19 pandemic has recently had an impact on the provision of health services, further bringing to the fore the role that mHealth apps can play in supporting public health [10,11]. Indeed, infection prevention and control measures have limited patients’ access to healthcare facilities and traditional communication channels with HCPs. In this challenging scenario, in order to support the continuity of care, a rapid expansion of telehealth and mHealth apps has been observed, especially for the management of long-term conditions such as chronic diseases [12].

However, the literature also indicates some limitations of the available mHealth apps and barriers to their adoption [13]. More in detail, research highlights that users or HCPs are scarcely engaged in the mHealth app development process [12]. Moreover, some authors highlight weaknesses perceived by end-users, such as the lack of disease-specific, cultural, and health literacy personalization (in other words, a good fit for a particular patient condition, different geographic, racial/ethnic, and socio-economic backgrounds) [13], limited accessibility, as well as the lack of evidence-based information conveyed by available mHealth apps [14]. The latter two aspects are particularly relevant if we consider that the decision of individuals to use mHealth services and tools on an ongoing basis is associated with the guarantee, reliability, and quality of the content of the service or tool itself [15].

The implementation of digital solutions that are effective in the management of health processes and satisfy end-users requires the application of user-centered design methods to understand the health information needs and preferences of the target population, including their preferences towards the content, functionalities and technical features of a specific tool such as a mHealth app [16].

To the best of our knowledge, to date, there are no studies addressing the adoption of a user-centered design methodology for developing and implementing a mHealth app to support family caregivers of children undergoing ENT surgery during the perioperative process.

Faced with this shortcoming, the general aim of the present study was to illustrate the user-centered design and development process of a mHealth app to support family caregivers of children undergoing tonsillectomy and/or adenoidectomy with or without tympanostomy tube insertion in a maternal and child health hospital during the perioperative process. The core of the participatory approach adopted in this process was the exploration of the needs, particularly informational/educational, and preferences of family caregivers (i.e., primary end-users) and HCPs (i.e., secondary end-users) towards the mHealth app to be developed. Informational support is indeed a crucial pillar of care for parents of pediatric patients: understanding and meeting their information needs allows for the provision of family-centered support as well as parental empowerment in caring for their child [6,14].

While mHealth apps are widely used to self-manage chronic conditions, they are not as frequently applied to support the management of the perioperative process associated with a surgical procedure such as ENT surgery. Our study focused specifically on the ENT surgery patient population because tonsillectomy and adenoidectomy with or without tympanostomy tube insertion are amongst the most frequently performed surgical procedures in the pediatric population [1,17]. Moreover, these surgeries have been shown to frequently generate challenges with home post-operative care and subsequent patient readmission to the emergency department [2]. Therefore, it is important to efficiently reach family caregivers of this patient population, including those with low health literacy, by offering them various educational strategies and tools, including mHealth apps.

Understanding, contextualizing, and responding to the needs and preferences of primary and secondary end-users is a critical process for the development and implementation of a mHealth app that both supports caregivers and integrates effectively into the care, management, and organizational processes of a hospital. The consideration of the needs and preferences of both primary and secondary end-users, from a participatory perspective, represents an added value in the mHealth app design, enabling the identification and incorporation of the points of view of these two categories of stakeholders in the process of developing and implementing the technological solution, thus increasing the likelihood that it will be acceptable.

### Research Questions and Study Aims

Starting from the above premises, our research activities were guided by the following research questions:

(1) What are the information/educational needs of family caregivers of pediatric ENT surgery patients accessing a maternal and child health hospital according to the caregivers themselves and the HCPs who care for these children?

(2) What are the most desirable content, functionalities, and technical features for a mHealth app to support caregivers of pediatric ENT patients during the perioperative process according to the primary end-users themselves and the secondary end-users?

(3) How do HCPs think a mHealth app could promote an efficient healthcare delivery to ENT pediatric patients and their family caregivers, integrating into the standard processes already existing in a maternal and child health hospital?

Considering the research questions above, the main three objectives (the first of which is divided into two sub-objectives) of the present study were as follows:

(1a) To explore the information/educational needs of family caregivers of pediatric ENT patients during the perioperative process;

(1b) To explore the experiences and perceptions of HCPs caring for children undergoing ENT surgery in relation to the information/educational needs of family caregivers and the main barriers to effective information/education at critical information moments during the ENT perioperative process;

(2) To identify primary and secondary end-users’ most desirable content, functionalities, and technical features of a mHealth app to support pediatric ENT patients and their family caregivers during the perioperative process;

(3) To explore how HCPs believe that a mHealth app could be useful for efficient healthcare to pediatric ENT patients and their family caregivers.

## 2. Methods

### 2.1. Study Design and Setting

A user-centered participatory design method was used. Following Schnall and colleagues [18], the steps of the study methodology were informed by the Information System Research (ISR) framework, which describes a process that can be effectively employed as a guide for the design of mHealth apps. The ISR framework consists of three cycles: the Relevance Cycle, in which the research setting (e.g., the environment and stakeholders) is explored; the Rigor Cycle, in which an evidence-based foundation for the process is created by evaluating theories and applying the acquired knowledge base; and the Design Cycle in which the results of the other cycles are used to inform the development of a product, in this case, a mHealth app to support family caregivers of children undergoing ENT surgery. As suggested by Schnall and colleagues [18] and described in the “Study design: structure and steps” section below, the cycles were conducted in an iterative process.

The study was carried out at a 136-bed maternal and child health hospital in northern Italy. The general pediatric surgical department of this hospital also admits children who need ENT surgery, and about 450 tonsillectomies or adenoidectomies are performed each year. There are 28 nurses, six nursing assistants, and, in addition to other medical specialists, five otolaryngologists working in the pediatric surgery department.

The user-centered design and development process of the mHealth app for family caregivers of pediatric ENT patients is an integral part of a larger project jointly developed by the hospital and Area Science Park, a public research institution located in Trieste (Italy). The aim was to design, develop, and test a digital ecosystem to support maternal and child health, with a particular focus on two categories of hospital end-users, namely expectant and new parents and their children in the first 1000 days of life and family caregivers of children undergoing ENT surgery. The digital ecosystem, the detailed description of which is not the subject of this paper, is designed to promote personalized education of users, thus responding to their specific information needs and increasing their empowerment.

The focus of the mHealth app development process, version 5.15.0, was on information content and some other mHealth app tabs and related functionalities (e.g., agenda and measures), while certain aesthetic features (e.g., colors and display of content) were not identified as priority elements to be customized. The mHealth app also has additional functionalities that could be implemented in the future, potentially providing a range of telehealth services to improve maternal and child healthcare, which will be the subject of further studies.

### 2.2. Study Design: Structure and Steps

The initial phase of the process involved assembling a committee with members possessing specific expertise in ENT surgery within the hospital, as well as individuals with specialized knowledge in e-health. The appointed committee comprised a total of 15 members (mean age 39.4; SD 9.3; 80% females) and included two ENT surgical nurses and one ENT physician with more than five years of experience in the ENT department; the ENT department director; the surgery head nurse; a nursing director; two surgical planning office nurses with experience in the ENT surgical ward; an operating room nurse; a research nurse; a physician PhD student; two engineers (main project managers); two designers of the company in charge of developing the mHealth app. Three members of the panel were appointed to keep track of the study’s progress and organize meetings. During each meeting, a member was in charge of taking field notes and then reporting a summary of the meeting by sending an e-mail to all the committee members, both those present and those who were absent for any reasons (e.g., organizational impediments, sick leave).

Alternating the Relevance and Rigor Cycles, the members of the committee were involved in a process that included the following steps:

(1) Identifying all the information/educational needs of family caregivers of pediatric ENT patients valuable for the development of a mHealth app intended to support them during the ENT perioperative process;

(2) To understand which topics are most difficult for family caregivers to grasp during the ENT perioperative process;

(3) To organize data collection to identify the most desirable content, functionalities, and technical features of a mHealth app to support family caregivers of children undergoing ENT surgery during the perioperative process through the consultation of primary and secondary end-users.

Later, through the Design Cycle:

(4) To define the content, functionalities, and technical features of the mHealth app to be developed in order to enhance its effectiveness in supporting family caregivers of pediatric ENT patients during the perioperative process.

The process of developing the mHealth application, in which the committee members played a crucial role, was aligned with the principles of ‘Slow Medicine’ [19], a systemic healthcare paradigm characterized by a step-by-step approach, attention to the environment (i.e., to the specific spatial and temporal context and to the expectations and perceptions of its actors), respect for patient values and preferences, and a commitment to providing appropriate care for all. In this sense, Slow Medicine [19] makes use of tools that encourage moments of discussion, participation, and collaborative designing between HCPs and citizens in favor of their health, thus promoting equitable care and improving the quality of life of citizens. In accordance with these basic principles, the committee involved in our study embraced the concept of ‘Slow design’ as a specific application of Slow Medicine, integrating it seamlessly into the adopted research methodology. So, while Slow Medicine serves as an overarching framework for healthcare, ‘Slow Design’ refers to the applicative translation of this general approach into the reflexive and participatory design methodology adopted to develop the mHealth app covered by this work. This methodology, harmonizing with the essence of Slow Medicine [19], aims to meet the preferences, expectations, and values of family caregivers regarding the information and education to be received throughout the ENT perioperative process before, during, and after hospitalization. Such an approach is functional for the design and development of a mHealth app that responds to the needs of primary and secondary end users and is, therefore, appropriate and effective.

**Figure 1:**
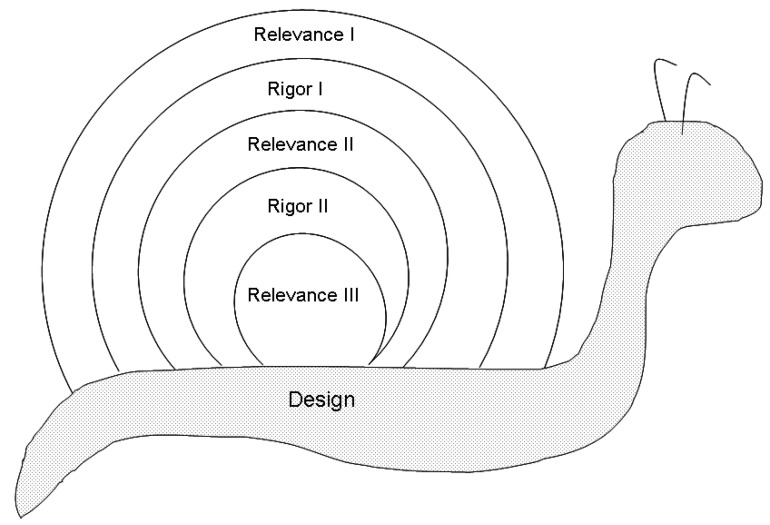
The steps of the ‘Slow design’ process followed in the mHealth app participatory development.

The six steps followed in the mHealth app ‘Slow design’ process are illustrated in Figure 1 and can be summarized as follows. (1) In the “Relevance I” step, six critical information/educational moments and related topics in the ENT perioperative process were identified; (2) In the “Rigor I” step, through a literature analysis, the educational topics related to pediatric ENT surgery that are critical for family caregivers were identified; (3) In the “Relevance II” step, the main knowledge gaps and barriers in the education of pediatric ENT patients’ family caregivers during the perioperative process were identified; (4) In the “Rigor II” step, primary (i.e., family caregivers) and secondary (i.e., HCPs) end-users’ preferences for the content, functionalities, and technical features of a mHealth app to support pediatric ENT patients’ family caregivers were identified through a literature analysis; (5) In the “Relevance III” step, the importance perceived by primary and secondary end-users of the content, functionalities, and technical features identified for the mHealth app was assessed; (6) In the “Design” step, the mHealth app was developed using the results of the other cycles and fine-tuning was performed by designers. These six steps are described in detail in the following sections.

### 2.3. Data Collection

The project started in January 2020. In October 2021, ethical approval for the data collection of the study was obtained from the Institutional Review Board of the hospital (IRB-BURLO 25/2021). Participation in the study was on a voluntary basis. Informed consent was obtained from each participant (i.e., family caregivers and HCPs) before data collection was conducted during the session III of the Relevance Cycle (described in detail below). To ensure confidentiality, the data collected were stored in a password-protected electronic folder. The anonymity of the participants was guaranteed.

Measures to prevent and control the spread of COVID-19 infection prevalent in the region where the maternal and child health hospital involved in the project was located at the time of the study were respected. To guarantee social distancing and encourage participation, the meetings with the panel of experts were organized mostly online through the Lifesize meeting platform or alternatively in a large meeting room in the hospital.

As anticipated above, the participatory design methodology that led to the development of the mHealth app to support family caregivers of pediatric ENT patients consisted of the six steps shown in Figure 1 and further described in detail below.

### 2.4. The Relevance Cycle (Session I: Identifying Critical Information/Educational Moments and Related Topics in the ENT Perioperative Process)

In the first session of the Relevance Cycle, the committee sought to better understand the hospital environment and the critical moments in the ENT perioperative process when information and education are provided by HCPs to family caregivers. These moments were defined by the committee members by examining the period from the first admission of the pediatric ENT patient and their caregiver to the hospital to the day of follow-up. Specifically, the following six critical information/educational moments were identified:

(1) the first inpatient ENT surgical consultation; (2) the phone call from the ENT surgical planning office to the primary caregiver to communicate the date of surgery and the list of documents to bring on the day of pre-admission consultations; (3) the day of pre-admission consultations when family caregivers are asked to bring the documents for hospital admission and nursing, and when the surgical and anesthesiological consultation and examination are performed; (4) the day of the family’s admission to the hospital and surgery; (5) discharge from the hospital and preparation for post-surgery care at home, usually on the same day surgery is performed (if no complications occur); (6) the follow-up visit in the hospital seven days after surgery.

Once the critical information/educational moments of the ENT perioperative process were defined, the expert committee members discussed identifying and listing all the topics that should be communicated to family caregivers of pediatric ENT patients at each of these moments. To do this, the experts also examined all the brochures and paper instructions that were routinely provided to these caregivers in the hospital.

### 2.5. The Rigor Cycle (Session I: Identifying Evidence-Based Educational Topics Critical for Family Caregivers of Pediatric ENT Patients)

For the sake of completeness, the committee members decided to identify other topics to be added to the list drawn up as a result of session I of the Relevance Cycle. For this purpose, following Schnall and colleagues [18], the expert panel identified two committee members who were appointed to conduct a literature analysis on the basis of their previous research experience in medicine (LB) and nursing (RD), respectively. In particular, topics relating to tonsillectomy and/or adenoidectomy with or without tympanostomy tubes insertion and family–centered care in the perioperative process, as well as those addressed in available scientific publications on educational tools related to ENT surgery in children were explored. The search was limited to peer-reviewed literature published from 2011 to 2021 in English or Italian language. The main search strings used to conduct the analysis were the following: “tonsillectomy” (OR “adenoidectomy” OR “tympanostomy”) AND “pediatric” (OR “paediatric”) AND “education” (OR “patient education” OR “caregiver education” OR “caregiver information” OR “perioperative education” OR “family-centered care”) AND “mHealth” (OR “mobile health” OR “mobile application”) OR “pamphlet” OR “booklet” OR “text messaging”. Electronic databases searched included Medline via Pubmed and CINAHL.

The key concepts identified through the literature analysis were then presented to the other members of the committee by the two experts responsible for the research. Some examples of the selected works that were considered by the committee to be particularly relevant for identifying critical topics for carers of pediatric ENT patients are Newton (2018) [20], Finestone (2019) [21], Yu and Kim (2019) [22], and Mendoza et al., (2021) [7].

### 2.6. The Relevance Cycle (Session II: Identifying the Main Knowledge Gaps and Barriers in Pediatric ENT Patients’ Family Caregivers Education during the Perioperative Period)

In order to optimize the mHealth app functionalities and considering that this digital tool was meant to meet the information needs of family caregivers but also facilitate and improve the efficiency and effectiveness of HCPs workflows in the perioperative process, the committee deemed it was appropriate to explore the topics that HCPs and surgical planning office staff felt were the most difficult for family caregivers to understand or put into practice at different moments of the ENT perioperative period and which impacted them in some way.

To this end, the committee members discussed the questions caregivers usually ask the hospital staff, the degree to which families are prepared when entering the hospital in terms of knowing what documents to bring or hygiene and dietary practices (e.g., fasting) before and after surgery, and the degree to which children know and understand the different stages of the perioperative process. The committee discussion revealed that the questions most frequently asked by caregivers concerned the length of hospital stay, waiting times for surgery and specialist visits, and the duration of the fasting of the child before and after surgery. Experts also described as critical the fact that, apart from the education provided by hospital staff, children are often not sufficiently prepared for surgery on the day of admission in terms of fasting, hygiene, or knowledge of the hospital stay and surgery, with potentially negative implications in terms of perioperative anxiety. Another crucial point that emerged among the experts concerned the fact that some parents purposely withhold information from children in an attempt to protect them from getting scared. The discussion also highlighted that some of the barriers encountered in sharing information with families stem from language and cultural differences and that caregivers sometimes acquire misleading information through personal searches on social media or the Internet. Session II of the Relevance Cycle, together with session I of the Relevance Cycle and session I of the Rigor Cycle previously described, helped to define the content for the mHealth app.

### 2.7. The Rigor Cycle (Session II: Identifying Primary and Secondary End-Users’ Preferences for the Content, Functionalities, and Technical Features of a mHealth App to Support Family Caregivers of Pediatric ENT Patients)

Another step that the committee members considered crucial was to understand the preferences of primary and secondary end users in terms of content, functionalities, and technical features, such as the quality of the information provided, personalization, and privacy features of a mHealth app to support caregivers of pediatric ENT patients. To do this, two researchers (LB and CDV) from the committee with previous research experience in the field of mHealth were appointed to perform a literature analysis on these topics through electronic databases such as Medline via Pubmed and CINAHL from 2011 to 2021, including only English or Italian language articles. The following search strings were used to perform the analysis: “user-centered design” OR “evaluation study” AND “mHealth” (OR “mobile health” OR “mHealth apps” OR “apps”) AND “usability” (OR “functionality” OR “acceptability” OR “desirable features” OR “preferred features” OR “patient perception” OR “privacy” OR “ethical issues”). As in the case of session I of the Rigor Cycle, identified sources of evidence were reported to other committee members and discussed. Some examples of findings considered particularly relevant by the committee are Brunelli et al. (2021) [23], Couture et al. (2018) [16], Gagnon et al. (2016) [24], Nouri et al. 2018) [8], and Vo (2021) [14].

### 2.8. The Relevance Cycle (Session III: Assessing the Importance Perceived by Primary and Secondary End-Users of the Content, Functionalities, and Technical Features Identified for the mHealth App)

In an iterative process within the Relevance and Rigor Cycles, the committee members developed a questionnaire targeting both primary (i.e., ENT patients’ family caregivers) and secondary (i.e., HCPs) end-users to probe the importance they perceive of a set of informative/educational content, functionalities, and technical features of a mHealth app that effectively support caregivers during the ENT perioperative process. Considering the barriers faced by caregivers in the perioperative period identified in session II of the Relevance Cycle, as well as the risks of misinformation that characterize digital societies and their potential impact on citizens’ health decision-making processes [25], the committee included items in the questionnaire aimed at exploring the information sources most commonly used by ENT patients’ caregivers. Additionally, the questionnaire sought to understand the improvements expected by primary and secondary users from the use of a specific mHealth app to support family caregivers during the ENT perioperative process.

The content validity of the questionnaire was evaluated by the committee members. They first assessed whether the items in the questionnaire adequately measured the construct intended to assess, whether the items were numerically sufficient to measure the domains of interest, and the order in which items were presented. Moreover, they evaluated if the items were written in a clear and easy-to-understand format [26]. Specifically, the two engineers and the two mHealth app designers (i.e., project managers) of the expert committee, who are not experts in the medical field, assessed the clarity of the items exploring the educational content of the mHealth app. Moreover, two nurses and two physicians from the expert committee assessed the clarity of the items, exploring the functionalities and technical features of the mHealth app. Finally, a small sample (five) of respondents belonging to the target population (i.e., family caregivers of pediatric ENT patients) further tested the questionnaire to indicate any unclear, ambiguous, or confusing items. Adjustments were made to the questionnaire until a unanimous consensus was reached within the committee.

The final version of the self-report questionnaire comprised 57 items. In particular, the items examining participants’ perceived importance of a specific mHealth app content, functionality, or technical feature had a 5-point Likert scale response option (from 0 = not important at all; 1 = not very important; 2 = fairly important; 3 = very important; 4 = absolutely essential). The items exploring the information sources most commonly used by primary users and the improvements expected from using the mHealth app required multiple-choice responses. After each questionnaire section, a free-text field was available for personal comments. The questionnaire also explored the socio-demographic characteristics of both primary and secondary end-users and, only in the case of HCPs, information on their professional profile and experience.

After receiving ethical approval, data collection started in January 2022 and ended in May 2022.

### 2.9. Participants of the Relevance Cycle (Session III)

Using purposive sampling based on reasoned selection and maximum variation, ENT department nurses, head nurses, physicians, and nurses from the surgical planning office who had more than six months of work experience in the pediatric surgery ENT department were recruited to participate in the cross-sectional study. In parallel, Italian-speaking family caregivers of children aged two to ten attending the ENT surgical department for tonsillectomy and/or adenoidectomy follow-up visits were included in the study using convenience sampling. Data collection from family caregivers took place in the surgical ward in the seating area near the visiting room where the follow-ups took place.

### 2.10. Data Analysis of the Relevance Cycle (Session III)

Descriptive statistics on sociodemographic and professional characteristics of participants (i.e., HCPs and family caregivers) were used (mean and percentage). Descriptive analysis was conducted for the ratings reported by HCPs and family caregivers, calculating the mean (M), standard deviation (SD), and frequency for the responses to each questionnaire item. To highlight the most important content as referred by participants, the three highest-scoring items of the questionnaire for each group of participants (i.e., HCPs and family caregivers) were identified and synthesized. Finally, a non-parametric Wilcoxon signed ranks test was performed to determine if there was a statistically significant difference (*p*-values < 0.05) in the questionnaire scores between the HCPs group and the family caregiver group.

### 2.11. Results of the Relevance Cycle (Session III)

A total of 24 HCPs and 25 family caregivers were enrolled in the study. Of the HCPs, 88% (*n* = 21) were nurses, and 53% (*n* = 13) had more than five years of experience in their role in the pediatric surgery ENT department. Considering the small sample size, age, and gender were not collected to maintain anonymity. Caregivers of children, on the other hand, had a mean age of 40 years (SD = 6.91). Among them, 8% (*n* = 2) were university graduates, and the rest had a lower education. Moreover, 12% (*n* = 3) of family caregivers were HCPs in a setting other than the maternal and child health hospital involved in the project, and 8% (*n* = 2) had previous experience with their child’s pediatric surgery.

Means and SD for the responses to each questionnaire item by HCPs and family caregivers, reflecting the importance given to the content, functionalities, and technical features of the proposed mHealth app, are presented in Table 1.

The items with the three highest mean scores recorded by family caregivers and HCPs were identified. The items that were prioritized by the caregivers mostly related to the content of the mHealth app. Specifically, in decreasing order of priority, they concern post-surgery pain management (M = 3.9; SD = 0.3), post-discharge mouth bleeding (M = 3.8; SD = 0.4), information about surgery and related risks during the pre-surgery phase (M = 3.7; SD = 0.9), post-discharge feeding and drinking (in terms of types of food and drink) (M = 3.7; SD = 0.6), post-discharge vomiting (M = 3.7; SD = 0.6) and post-discharge pain (M = 3.7; SD = 0.6). Only two technical features of the mHealth app were prioritized by caregivers, namely the extent to which the mHealth app implements intuitive and predictable navigation patterns (M = 3.7; SD = 0.6) and the presence of content validated by an institutional source (M = 3.7; SD = 0.6).

As with family caregivers, also in the case of HCPs, the questionnaire items rated with a higher score related to the content of the mHealth app. In decreasing order of priority, they related in particular to the child’s preparation/information for hospitalization/surgery in the pre-operative phase (M = 3.7; SD = 0.6), to eating and drinking before surgery (in relation to the type of food and drink) (M = 3.7; SD = 0.5), on eating and drinking after surgery (in relation to timing) (M = 3.7; SD = 0.5), on information about the surgery and the associated risks in the pre-operative phase (M = 3.6; SD = 0.7), eating and drinking after surgery (in relation to the type of food and drink) (M = 3.6; SD = 0.5), pain management after surgery (M = 3.6; SD = 0.6), eating and drinking after discharge (in relation to the type of food and drink) (M = 3.5; SD = 0.7), pain management after discharge (M = 3.5; SD = 0.7) and bleeding from the mouth after discharge (M = 3.5; SD = 1.0). The only technical feature that emerged as a priority for HCPs was the extent to which the mHealth app implements intuitive and predictable navigation patterns (M = 3.5; SD = 0.7). The aspects that both caregivers and HCPs consider most important can, therefore, be summarized as follows: information about surgery and related risks, content on pre- and post-surgery and post-discharge feeding and drinking, content on post-surgery and post-discharge pain management, content on post-discharge mouth bleeding and the extent to which the mHealth app implements intuitive and predictable navigation patterns (see details in Table 1).

However, the results also show statistically significant differences between the two groups with reference to the importance attributed to certain content, functionalities, and technical features of the mHealth app, including the declaration (through the provision of specific references) of the scientific accountability of the content provided through the app (M caregivers = 2.9; M HCPs = 1.7; *p*-value = 0.002), the presence of social mechanisms that allow the user to interact with healthcare staff (e.g., community, forum, chat) (M caregivers = 3.4; M HCPs = 2.4; *p*-value = 0.004) and information on how to help the child in case of voice alterations, bad breath or white/yellow spots in the throat (M caregivers = 3.6; M HCPs = 2.8; *p*-value = 0.004). Other significant differences between the two groups are presented in Table 1.

No participant left additional comments in the space for free text in the questionnaire.

### 2.12. Discussion of the Relevance Cycle (Session III)

Results show how both family caregivers and HCPs consider the information/educational content of the mHealth app more relevant than its functionalities and technical features. Moreover, the priority assessments of the two groups were similar, suggesting an alignment—encouraged by a growing organizational health literacy—between the ability of HCPs to grasp families’ needs and concerns and the ability of caregivers to identify at an early stage what is a priority for their children’s ENT surgical pathway and what requires more attention [27]. The topics of greatest interest for caregivers are in line with the literature and include information on pain control, diet, surgical procedures, and their related risks [28,29]. Interestingly, most of the content deemed particularly important by caregivers concerned information useful for post-surgery management at home after discharge, including feeding and drinking, as well as possible problems such as bleeding, vomiting, or pain. In this context, the literature available to date suggests that family caregivers following ENT surgery rely heavily on the management of their children’s post-operative recovery at home and feel somewhat responsible for the complicated course of recovery or unexpected outcomes, highlighting the need for further support [30].

In our study, HCPs are instead equally interested in all phases of the ENT perioperative process. Particular importance was recognized by HCPs on informing and adequately preparing the child for hospitalization and surgery, which was not considered equally relevant by family caregivers, confirming the barriers in the education process during the ENT perioperative period highlighted by the expert panel during session II of the Relevance Cycle. The literature suggests that caregivers often do not adequately inform their children about hospitalization and the surgical process because they are worried that such information may frighten them. However, research has proven that age-appropriate education and the adoption of child-centered care are critical steps for the active participation of children in the care process by respecting and integrating their perspectives and needs, which have a positive impact on clinical practice [31]. Previous experiences also show that providing information on hospitalization and surgery to children reduces their pre-operative concerns [32]. In light of these findings, it seems crucial that the process of developing a mHealth app to support pediatric ENT patients’ family caregivers also takes into account the importance of sharing some information with the children using ad hoc methods and times.

The interest shared by caregivers and HCPs towards the mHealth app’s ability to implement intuitive and predictable navigation patterns is in line with previous literature [14,24]. Moreover, one of the priorities for family caregivers is that the mHealth app contains a declaration of the scientific responsibility of the provided information/educational content that guarantees its validity. This point is consistent with the literature suggesting that users are sometimes skeptical of the mHealth apps on the market because there is no proof that the information conveyed comes from a reliable source or is evidence-based [16].

Finally, the priority given by family caregivers to the presence of social mechanisms allowing the user to exchange and interact with healthcare staff does not meet the ratings of HCPs. This point may reflect the widespread perception among HCPs that mHealth tools can be invasive and intrusive and that their use may increase their workload [24].

### 2.13. The Design Cycle (the Development of the mHealth App Using the Results of the Other Cycles and Fine-Tuning Stage by the Designers)

The results of the literature analysis performed in sessions I and II of the Rigor Cycle and the results of the analysis of the questionnaires evaluated in session III of the Relevance Cycle allowed us to define a list of content, functionalities, and technical features that were considered in the process of mHealth app development, paying particular attention to the content deemed important by both family caregivers and HCPs. In this context, for example, it became clear that the content originally designed for the mHealth app to inform/prepare the child for hospitalization and surgery needed to be improved. Therefore, new documents were drawn up to address this critical area, such as two illustrated files explaining the surgery pathway and anesthesia to young children, content that educates caregivers on the importance of involving children in their care process, and some tips for parents on how to prepare their child for hospitalization and surgery. Likewise, it was decided to devote a considerable part of the mHealth app content to suggestions for caregivers on how to deal with post-discharge potential problems and complications at home.

A multi-professional group (i.e., a research nurse, a surgical head nurse, a nurse from the public relations office, and a physician from the ENT department) was then appointed by the expert committee members to work on the definition of the information/educational content for the mHealth app. The content was developed by drawing on the scientific literature previously examined in sessions I and II of the Rigor Cycle, also taking into account the brochures and paper instructions that are usually given to families during hospital stays, reviewing and updating them as needed. The content was reported in Word files. Once the drafting stage was completed, the files were directed to the other HCPs of the committee to be checked and edited as necessary. This process continued until a final consensus was reached on the content of each document. A synoptic overview of the information/educational content provided by the mHealth app and the relative temporal phases of the ENT perioperative process to which they refer is shown in Table 2.

To further tailor the mHealth app content to the recommended reading level for the information needs of public healthcare and primary care end-users [33], a team of external communication experts was involved in the mHealth app development process to edit the prepared information/educational content in order to make it equally accessible to a general public audience.

Then, the developed content, together with the list of the mHealth app functionalities and technical features previously identified, was discussed with two designers from the selected company in charge of developing the mHealth app. In line with the adopted Slow design, it was determined that the mHealth app would provide personalized education to primary end-users during the ENT perioperative process, directing their attention and interest to what is most relevant and appropriate to their needs, at each stage of the ENT surgical pathway. To encourage user compliance, the family caregiver would receive a notification on their smartphone as they approach the scheduled information content, reminding them of the upcoming information/educational activity. For example, notifications are sent to family caregivers the day before the surgery to remind them to take care of the child’s hygiene and to bring the child’s documents with them on the day of admission to the hospital. Once the prototype of the mHealth app had been developed, all members of the committee were given a password to access it to examine and test the tool. After the trial period, the experts provided their evaluations of the tool by answering a series of questions aimed at investigating the presence or absence of certain functionalities and technical features (selected on the basis of the results of session II of the Rigor Cycle and session III of the Relevance Cycle) in the mHealth app prototype. They also completed the Mobile Application Rating Scale (MARS) [34], a structured questionnaire used to evaluate mHealth app quality, considering four dimensions of objective app quality, namely engagement, functionality, aesthetics, and information quality. The subjective app quality subscale and perceived impact section of the MARS were also completed. The evaluators then sent their answers by email, together with any suggestions for the improvement of the mHealth app prototype, to a committee member who analyzed and summarized the findings.

On the basis of priorities resulting from expert evaluations, a list of indications needed to improve the mHealth app prototype was drafted and unanimously approved by the committee members. Proposals for implementation of the mHealth app included the ability to switch the screen orientation from vertical to horizontal view to facilitate the reading of certain long information/educational documents, the ability to display the whole week and month in the calendar to facilitate user exploration (in fact, the mHealth app prototype only showed a daily visualization), improving the design of the user interface by including a homepage that unifies and organizes the different tabs of the mHealth app so that not too many screens were needed to access useful information and switch from one tab to another.

The proposals for implementation were then forwarded to the designers of the company in charge of developing the mHealth app, and the prototype was refined, addressing the main gaps or critical issues identified by the committee. Thus, based on the overall results of the Design Cycle, a final product was finally available for experimental testing.

**Figure 2:**
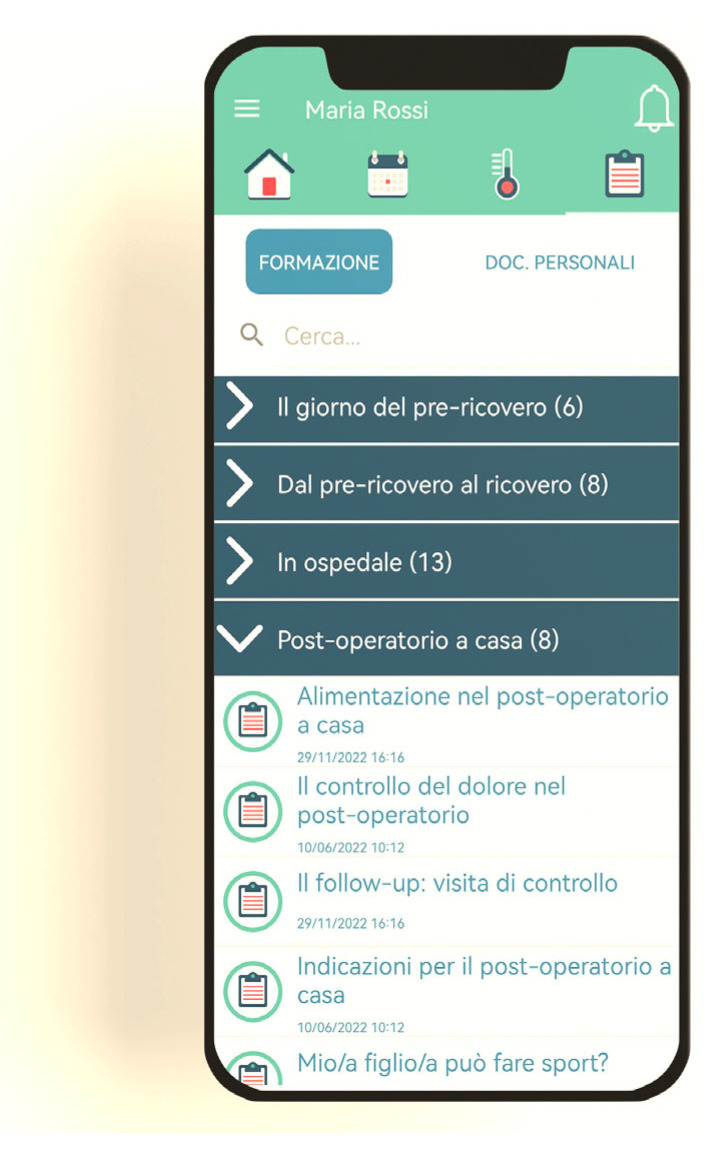
“Training” section of the mHealth app. The figure is a reproduction of the original in the article by Dobrina and colleagues [27]. Permission for use has been obtained from Springer Nature (License Number 5686551351311; License date: 12 December 2023).

The “Training” section of the mHealth app “*AreaBurlo*”, contains all the information/educational contents (i.e., text documents, sometimes also integrated with images) relating to the ENT perioperative process and available to users (i.e., family caregivers) at any time, is illustrated in the screenshot shown in Figure 2. In particular, the upper area of the app, in light green, displays the available app tabs. For example, by clicking on the house icon, the user accesses the home page of the app, while clicking on the notebook icon, the user accesses the “Training” section (that can be translated in Italian as “Formazione”) In the central part of the screenshot, in dark blue, the different phases of the ENT perioperative process are indicated with their respective titles (for example, “Il giorno del prericovero” which translated into English would be “The day of pre-admission consultations” or “Post-operatorio a casa” that is the Italian version of “The post-operative care at home”). Each phase of the surgical process corresponds to an information section (like a folder) containing various documents (i.e., information/educational contents). Therefore, as visible in the lower part of the screenshot reported in Figure 2, by clicking on the title of each phase of the ENT perioperative process, it is possible to view and read the documents (shown in white) referred to that phase. For example, clicking on the title “Post-operatorio a casa” takes the user accesses to the document “Alimentazione nel post-operatorio a casa”, which can be translated into English as “Nutrition at home after surgery”. Although the “Training” section is a core of the “*AreaBurlo*” mHealth app (the app development process actually focused primarily on information content), this tool does more than just convey information content to users. However, as anticipated in the “Study design and method” section of the paper, the other app functionalities (e.g., the previously mentioned ability to send notifications to users) and features are not extensively covered in this work.

A poll was then held involving 164 hospitals’ HCPs to define the name of the developed mHealth app. Among the five options proposed by the expert committee, the winning name was “*AreaBurlo*”. “*AreaBurlo*” combines a part of the names of the two institutions that jointly developed the project within which this study falls, namely “*Area* Science Park” (the public research institution) and “*Burlo* Garofolo” (the maternal and child health hospital). Moreover, considering another point of view, the name “*AreaBurlo*” indicates a digital “*area*” (i.e., a repository) specifically dedicated to contents relating to the IRCSS Burlo Garofolo.

A randomized controlled trial to evaluate the effectiveness of the mHealth app developed to support pediatric ENT patients’ family caregivers compared to standard care (i.e., standard information and education about the ENT perioperative period provided to caregivers orally by HCPs or through brochures) is underway [35].

## 3. Discussion

Nowadays, the lack of resources in healthcare systems cannot guarantee adequate services and time to meet all the needs and preferences of patients and their family caregivers, even in pediatric settings [30]. Within this context, there is a growing and increasingly widespread impetus from the mHealth app industry for the development of systems and tools to support HCPs to optimize the services provided to users and improve customer satisfaction [8]. However, authors in the literature warn that the quality and reliability of the mHealth apps currently available on the market should be evaluated with caution [36]. Today, to ensure high-quality and safe care through the support of mHealth apps, the adoption of a patient-centered approach and an evidence-based design is indeed essential.

In our study, triangulation of data sources, through the collection of information from experts in e-health and mHealth apps to improve healthcare, family caregivers of ENT surgery patients and HCPs from different professions and areas of the ENT department, including hospital management and administration, ensured a participatory Slow design approach that enabled the development of a user-centered and customized mHealth app that meets the needs and preferences of end-users in a pediatric setting. The development and implementation of such mHealth apps are expressions of collaboration between HCPs and citizens, fostering a more patient-centered healthcare system.

The content provided by the mHealth app is not only tailored to the specific health needs related to the perioperative ENT process but also meets the information needs of a wider audience that is based on scientific and grey literature [14].

In terms of implementing participatory processes, our findings also highlight the importance of increasing the involvement of both pediatric patients and their caregivers in healthcare through interventions that explore and encourage communication and sharing of needs, raise awareness, and educate families. Family caregivers can, therefore, become privileged vehicles of information to their children, increasing their preparation for hospitalization and surgery.

These process features not only draw from the principles of Slow Medicine [17] but also adapt them into a Slow design framework specific to the development of the mHealth app described in the present paper. This fit enhances the application of Slow Medicine principles in the context of the design (and co-design) and development of technological solutions like the one that is the subject of our work. In particular, Slow Medicine’s emphasis on gradualness influenced the adoption of the step-by-step design methodology applied in the participatory development of the mHealth app. This deliberate pace allowed us to thoroughly integrate end-user values and preferences into the design, ensuring that the final product was reflective of the diverse needs and expectations of the target population. The development and future implementation of such a mHealth app, which provides accessible information and educational resources to primary end-users, emphasizes the role of mHealth tools in empowering citizens to take control of their health and well-being, which can have a positive impact on improving health-related decision-making processes and health outcomes [14]. Moreover, the potential reduction in unnecessary hospital visits and the improved health-related decision-making, as facilitated by the mHealth app, have direct implications for resource allocation and public health spending [14].

Furthermore, the analyzed participatory approach has led to the creation of a valuable tool that goes beyond the confines of its original development setting. Its core features and content can be readily applied and transferred to other healthcare facilities in Italy and worldwide through a process of adaptation and customization. Adenoidectomy and tonsillectomy are indeed very common surgical procedures, and the challenges faced by family caregivers of pediatric patients undergoing these surgeries are universal. Moreover, by translating the mHealth app content into different languages and adapting it according to the internal regulations and procedures of local healthcare systems, it can benefit family caregivers, pediatric patients, and healthcare processes in various international settings. Overall, these aspects make the developed app a potentially scalable and useful tool in very different contexts from a linguistic, cultural, regulatory, and procedural point of view.

Furthermore, the participatory design methodology adopted to develop the “*AreaBurlo*” mHealth app could be replicated in other pediatric medical specialties and clinical settings, acting as a trailblazer for the development of similar tools to support family caregivers. If, in fact, the contents of this app are obviously specific and targeted with respect to the ENT perioperative process, the different steps of the ‘Slow design’ process followed in the mHealth app participatory development can also be applied in favor of other clinical processes and paths, in relation to the different characteristics, temporal phases, and care needs that characterize each path. This participatory design methodology, which reflects the principles of Slow Medicine, can also be profitably used outside the pediatric field, thus showing potential for very broad and transversal scalability and applicability to numerous categories of patients. Finally, the adoption of a standardized methodological framework, such as the ISR [16], that guided the development of the mHealth app described in this paper adds value to the overall study design.

## 4. Limitations

In addition to the research framework adopted to guide the study process, a more structured method of developing consensus among the committee members within the research cycles, for example, a Delphi methodology [37], would have improved the quality and reliability of results. Moreover, the participation of family caregivers in data collection in session III of the Relevance Cycle was poor. However, this low adherence can be explained in light of the restrictions imposed during the COVID-19 pandemic. Caregivers were indeed admitted to the hospital only strictly for the time of scheduled visits to avoid overcrowding, thus limiting their time for participation in the study. Moreover, only one parent per child was admitted for follow-up visits, and considering that children undergoing ENT surgery are between the ages of two and ten, caregivers sometimes refused to participate because they had to take care of their children. Furthermore, compared to the standard practice in pandemic-free periods, few families decided to accept the follow-up visit to the hospital, preferring to refer to their family pediatrician, especially those who came from out of town. Finally, hospital staff shortage due to COVID-19 infection limited the availability of nurses for the research data collection, presumably impacting the number of study participants. Despite the unanticipated impacts of the pandemic, the timetable for the development of the mHealth app still had to be respected for the agreement signed by the project partners (for further information, see the “Funding” section). Data collection was therefore interrupted in May 2022 to allow the developing phase of the mHealth app to respect the schedule.

Finally, children, i.e., those who personally experience and face the ENT perioperative process, were not included in the study. However, considering that children undergoing tonsillectomy or adenotonsillectomy usually are between the ages of two and ten years old and are often pre-schoolers, a self-report assessment questionnaire such as the one administered to family caregivers and HCPs in the present study would have been ineffective with such young children. Moreover, given the wide range of their ages, different data collection methods and tools (i.e., tailored to be age-appropriate) would have been necessary, leading to potentially biased measures. Future studies could, however, consider including a sample of children as homogeneous in terms of age alongside caregivers and HCPs since the point of view of pediatric patients is central to implementing healthcare pathways that are attentive and appropriate to their needs in accordance with a patient-centered approach, practice, and care.

## 5. Conclusions

Our study describes the design and development process of a mHealth app through the ISR standardized methodological framework to support family caregivers of pediatric ENT patients undergoing tonsillectomy or adenotonsillectomy before, during, and after hospitalization and surgery. The involvement of caregivers (i.e., primary end-users) and HCPs (i.e., secondary end-users) in the various stages of development of the mHealth app as well as the inclusion in the app of evidence-based educational content has the potential to improve the organizational requirements of healthcare facilities, enhancing the delivery of appropriate and effective care. Such a mHealth app, even in an offline format, can indeed indirectly ensure more equitable access to care by representing a supportive information tool for families in a healthcare context characterized by a short hospital stay and limited time that HCPs can devote to providing targeted and personalized education. Moreover, the study contributes theoretically by introducing the concept of “Slow Design” within the framework of mHealth app development. This theoretical approach, stemming from the Slow Medicine paradigm, introduces a new perspective to technology design in healthcare.

## Figures and Tables

**Table 1:** Descriptive analysis for the ratings reported by family caregivers and healthcare providers, calculating means and standard deviations (SD) for the answers to each item.

Domain	Item	Family Caregivers (*n* = 25)		Healthcare Providers (*n* = 24)		Wilcoxon *p*-Value
		Mean	SD	Mean	SD	
Before surgery	Information about tonsils or adenoids or effusive otitis media	3.4	0.7	2.9	0.9	* 0.047
	Information about surgery and related risks	3.7	0.9	3.6	0.7	0.783
	Information about anesthesia and related risks	3.5	0.9	3.1	1.4	0.654
	Information about the medications the child can take before hospitalization	3.2	1.0	3.1	1.1	0.975
	Information about how to prepare/inform the child for hospitalization/surgery	3.3	1.0	3.7	0.6	* 0.045
	Information about the documents needed for the surgery	2.8	1.0	3.3	1.0	* 0.022
	Information about the need to pay a sum for procedures	2.4	1.2	2.0	1.3	0.464
	Information about the waiting time for the surgery	3.3	0.7	2.6	1.3	* 0.054
	Information about the map of the hospital	2.7	1.0	2.6	1.2	0.840
	Information about what the child can drink/eat before surgery	3.5	0.7	3.7	0.5	0.496
	Information about what parents can do to help their child when entering the operating theatre together	3.5	0.7	3.3	1.0	0.616
After surgery	Information about when the child will be able to drink/eat?	3.6	0.5	3.7	0.5	0.207
	Information about what the child will be able to drink/eat?	3.6	0.5	3.6	0.5	0.945
	Information about when the child will be able to remove the needle-cannula and intravenous fluids?	3.0	1.0	3.1	1.0	0.553
	Information about pain control	3.9	0.3	3.6	0.6	0.106
At home after discharge	Information about what will be better for the child to drink/eat?	3.7	0.6	3.5	0.7	0.434
	Information about whether the child may shower or brush his/her teeth	3.2	1.0	2.8	1.0	0.248
	Information about when the child will be able to go back to school	3.5	0.7	3.1	0.9	0.106
	Information about when the child will be able to return to sports activities	3.1	1.1	3.0	0.8	0.669
	Information about when the child will be able to go to the beach/pool	3.0	1.0	2.8	0.9	0.542
	Information about how to help the child in case of eat/drink refusal	3.6	0.6	3.2	1.0	0.277
	Information about how to help the child in case of vomiting	3.7	0.6	2.9	1.2	* 0.023
	Information about how to help the child in case of fever	3.5	0.6	3.1	1.1	0.326
	Information about how to help the child in case of pain despite the medications prescribed	3.7	0.6	3.5	0.7	0.365
	Information about how to help the child in case of bleeding from the mouth	3.8	0.4	3.5	1.0	0.454
	Information about whether the child can blow his/her nose and how to do it	3.3	0.8	3.4	1.0	0.441
	Information about how to help the child in case of voice alterations or bad breath or white/yellow spots in the throat	3.6	0.6	2.8	1.1	** 0.004
Reminders and push notifications	App’s ability to set reminders for medical appointments (e.g., follow-up visit)	3.5	0.8	3.3	1.0	0.477
	App’s ability to schedule reminders for routine activities (e.g., drugs administration)	3.1	0.9	2.5	1.1	0.078
Notes and records	App’s ability to record the problems after discharge at home (e.g., pain, vomiting, fever)	3.3	0.9	2.7	0.9	* 0.018
	App’s ability to record physiological values of the child (e.g., temperature)	2.8	0.9	2.4	0.9	0.119
	App’s ability to record routine activities of the child (e.g., drinking, eating, sleeping patterns/times, urinary output)	2.7	1.0	2.4	0.8	0.385
	App’s ability to record the medications taken by the child	2.6	0.9	2.3	1.0	0.339
Social support	Integration of the app with social networks (e.g., Facebook, Twitter)	1.6	1.2	1.4	1.3	0.726
	Presence of a FAQ (frequently asked questions) page in the app	2.7	1.2	2.8	1.1	0.461
	Presence of social mechanisms allowing the user to interact with each other and share experiences (e.g., community, forum, and chat)	2.5	1.4	1.7	1.1	* 0.028
	Presence of social mechanisms allowing the user to interact with healthcare staff (e.g., community, forum, and chat)	3.4	1.0	2.4	1.2	** 0.004
App technical features	Authentication request to the user	2.5	1.4	2.5	1.2	0.883
	Presence of a privacy policy in the app	2.6	1.3	3.1	1.4	* 0.056
	Ability for the user to access all app content for free (without any payment)	3.5	1.0	3.4	1.0	0.718
	Access to full app usage based on specific inclusion criteria (e.g., national health service card, place of living, and authorization by a health professional)	2.5	1.3	2.3	1.4	0.845
	Presence of references about the contents provided through the app	2.2	1.1	2.1	1.1	0.725
	Presence of a glossary of the most used medical terms	2.9	1.0	2.5	1.1	0.259
	Declaration (through the provision of specific references) of the scientific responsibility of the contents provided through the app	2.9	0.9	1.7	1.3	** 0.002
	Possibility to back-up/restore data within the app	2.7	1.1	2.0	1.2	0.111
	Possibility to download data collected through the app	2.6	1.0	2.4	1.1	0.623
	App’s ability to book visits and checkups	3.6	0.8	2.7	1.3	* 0.032
	App’s ability to update users’ account preferences	2.9	1.2	2.3	1.3	0.088
	Use of a simple, informal, and friendly tone by the app	3.1	0.8	3.0	1.1	0.593
	App’s ability to adapt to screen orientation (both portrait and landscape)	2.4	1.3	2.4	1.3	0.771
	App’s ability to learn user’s preferences over time	2.7	1.2	2.2	1.3	0.146
	App’s ability to implement intuitive and predictable navigation patterns	3.7	0.6	3.5	0.7	0.823
	Presence of app contents validated by an institutional source (local, regional, or national)	3.7	0.6	3.2	0.7	0.076
	Presence of certification of the app as a medical device according to Italian law	3.0	0.9	2.4	1.2	0.193
	App’s ability to provide contents through different ways (e.g., text, video, audio)	3.0	1.0	2.7	0.8	0.157
	App’s ability to ask about user satisfaction	2.4	1.1	3.3	0.9	* 0.024

Legend: SD: standard deviation; ** *p* ≤ 0.01 and * *p* ≤ 0.05.

**Table 2:** A synoptic overview of the information/education content provided by the mHealth app and the relative temporal phases of the ENT perioperative process to which they refer.

Temporal Phase of the Ent Perioperative Process	Information/Education Content	
	Topics	Document Title
From the first inpatient ENT surgical visit to the pre-admission consultation day	Information on pre-admission consultation day	The pre-hospitalization day
	Information on how admission and surgery will be planned	Ordinary hospitalization, day surgery and waiting times for surgery
	Information on tonsillectomy or adenotonsillectomy surgery procedure	Information for caregivers of children undergoing tonsillectomy or adenotonsillectomy surgery
	Information on tympanostomy surgery procedure	Information for caregivers of children undergoing tympanostomy
	Information on the possible types of anesthesia	Anesthesia: how is it done and related risks
	Example of an informed consent form for anesthesia	Anesthesia techniques: informed consent
	The hospitals’ contact information in case of need before hospitalization and surgery	Are you waiting for surgery and do you need to contact us?
From pre-admission consultation day to surgery	Help family members to organize for hospitalization	Things to bring to the hospital on the day of hospital admission and surgery
	Advice to caregivers on preparing their child for hospitalization, surgery, and participation in the perioperative pathway	How to prepare my child for visits, hospitalization and surgery
	Explains caregivers how to prepare the child for SARS-CoV-2 nasal swab testing	Swab testing for SARS-CoV-2 screening
	Help family members to organize for hospitalization	Things to bring to the hospital on the day of hospital admission and surgery
	Information on how the day of hospital admission and surgery is organized	The day of hospital admission and surgery: how the day is organized and what will happen
	Illustrated booklet explaining hospitalization and surgery to children	“The operating moon”
	Illustrated booklet explaining anesthesia to children	“The doctor who gifts dreams”
In hospital, the day of surgery	Help the family to navigate the hospital	The day of hospital admission: reception and surgery ward—where and when
	Advice on fasting before surgery	The importance of fasting before surgery
	The surgery path	Illustrated map of the path from the surgery ward to the operating theatre
	Information about post-surgery (e.g., procedures, fasting, potential problems)	Information about post-surgery in the surgery ward
	Information about how pain control is guaranteed	Pain control after surgery
	Explain how and when hospital discharge is organized	Information on hospital discharge
After discharge: post-surgery at home	Help caregivers manage post-operative general issues and potential problems at home	Directions for post-operative care at home
	Pain: brief practical advice	What to do if he/she has pain
	Hemorrhage: brief practical advice	What to do if he/she is bleeding from the mouth
	Fever: brief practical advice	What to do if he/she has fever
	Nausea or vomit: brief practical advice	What to do if he/she has nausea or vomit
	Diet: brief practical advice	What to do if he/she doesn’t eat or drink
	Voice: explains why children may have a different voice after surgery	What to do if he/she has a different voice
	Breath: explains why children may have bad breath after surgery	What to do if he/she has a bad breath
	Snoring: explains why children may snore after surgery	What to do if he/she snores
	Trans tympanic tube management: Brief practical advice	What to do if the trans tympanic tube is expelled prematurely
	Follow up visit: Information on the need of visit and what will happen	Information on follow-up visit
	Hygiene: brief practical advice	Can he/she have a shower/bath/wash teeth?
	Diet: brief practical advice	What can he/she eat or drink?
	Sport: brief practical advice	Can he/she practice sport?
General information on IRCCS Burlo Garofolo	Hospital’s service charter	The history of the maternal and child health hospital
	Hospital’s service charter	The mission of the hospital
	Hospital’s service charter	Organigram
	Hospital’s service charter	Where the hospital is and possible parking
	Hospital’s service charter	The hospital’s map
	Hospital’s service charter	The colors of our uniforms
	Hospital’s service charter	The pediatric surgery department
	Hospital’s service charter	Pictures of staff of the surgical ward and of the operating theatre
	Hospital’s service charter	Meal times and activities
	Hospital’s service charter	How and where to request a copy of the medical records
	Hospital’s service charter	COVID-19 infection prevention control measures and access to the hospital
	Hospital’s service charter	The hospital cafeteria and canteen service

## Data Availability

Data are contained within the article.
